# Complexity of Infection and Genetic Diversity in Cambodian *Plasmodium vivax*

**DOI:** 10.1371/journal.pntd.0004526

**Published:** 2016-03-28

**Authors:** Lindsey R. Friedrich, Jean Popovici, Saorin Kim, Lek Dysoley, Peter A. Zimmerman, Didier Menard, David Serre

**Affiliations:** 1 Genomic Medicine Institute, Cleveland Clinic Lerner Research Institute, Cleveland, Ohio, United States of America; 2 Unite d’Epidemiologie Moleculaire, Institut Pasteur du Cambodge, Phnom Penh, Cambodia; 3 National Center for Parasitology, Entomology and Malaria Control, Phnom Penh, Cambodia; 4 Center for Global Health and Diseases, Case Western Reserve University, Cleveland, Ohio, United States of America; New York University, UNITED STATES

## Abstract

**Background:**

*Plasmodium vivax* is the most widely distributed human malaria parasite with 2.9 billion people living in endemic areas. Despite intensive malaria control efforts, the proportion of cases attributed to *P*. *vivax* is increasing in many countries. Genetic analyses of the parasite population and its dynamics could provide an assessment of the efficacy of control efforts, but, unfortunately, these studies are limited in *P*. *vivax* by the lack of informative markers and high-throughput genotyping methods.

**Methodology/Principal Findings:**

We developed a sequencing-based assay to simultaneously genotype more than 100 SNPs and applied this approach to ~500 *P*. *vivax*-infected individuals recruited across nine locations in Cambodia between 2004 and 2013. Our analyses showed that the vast majority of infections are polyclonal (92%) and that *P*. *vivax* displays high genetic diversity in Cambodia without apparent geographic stratification. Interestingly, our analyses also revealed that the proportion of monoclonal infections significantly increased between 2004 and 2013, possibly suggesting that malaria control strategies in Cambodia may be successfully affecting the parasite population.

**Conclusions/Significance:**

Our findings demonstrate that this high-throughput genotyping assay is efficient in characterizing *P*. *vivax* diversity and can provide valuable insights to assess the efficacy of malaria elimination programs or to monitor the spread of specific parasites.

## Author Summary

*Plasmodium vivax* is responsible for most malaria cases outside Africa but remains poorly understood. Here we describe a high-throughput assay that enables genotyping more than 100 SNPs in 96 samples simultaneously. We applied this assay to characterize *P*. *vivax* genetic diversity in ~500 individual infections collected throughout Cambodia and including multiple time points. Our analyses revealed that the Cambodian *P*. *vivax* population is very diverse genetically but geographically homogenous, without any obvious boundaries. We also observed that almost all infected individuals carried more than one *P*. *vivax* parasite, though the proportion of single parasite infections tends to increase in recent years. Our study illustrates how genetic information can be generated to monitor temporal and geographical variations in parasite populations and help assess the efficacy of malaria control programs.

## Introduction

*Plasmodium vivax* is the most widely distributed human malaria parasite, threatening almost 40% of the world population [1]. It is the major cause of malaria outside of Africa, with up to 390 million clinical infections each year, and is responsible for long-term chronic illness that has dramatic consequences for the health and economy of endemic regions. Despite the implementation of extensive control efforts, the proportion of cases attributed to *P*. *vivax* has significantly increased over time compared to *P*. *falciparum* [2]. For example, in Cambodia the malaria control activities in the last ten years–extended access to insecticide treated bed nets, distribution of rapid diagnostic tests (RDTs) to health facilities, switch to dihydroartemisinin-piperaquine as first line therapy for treatment of uncomplicated malaria–have led to a 50% decrease in malaria cases (from 113,855 cases in 2004 to 56,271 cases according to data from the Ministry of Health). However, these measures disproportionally affected falciparum malaria: for the first time in 2011, the number of *P*. *vivax* infections (42,901) became higher than the number of *P*. *falciparum* infections (33,326) and by 2014, *P*. *vivax* was detectable in 78% of the malaria cases (46% of mono-infections and 32% of mixed infections). In order to effectively design elimination strategies to combat *P*. *vivax*, it is essential that we better understand how the parasite population is changing in response to control measures. This is particularly important for *P*. *vivax* since our inability to culture the parasite *in vitro* [3] complicates measuring the efficacy of antimalarial drugs in the population (though *ex vivo* studies can partially overcome this limitation [4–6]). Unfortunately, the lack of an *in vitro* culture system also limits the amount of genetic and genomic data available for *P*. *vivax*. Most population genetic studies in *P*. *vivax* have relied on small numbers of microsatellite markers (e.g., see [7–9]) or single nucleotide polymorphisms (SNPs) [10,11] or targeted a few protein coding genes [12]. These studies therefore have limited power to identify population structure (that typically requires many unlinked markers) or to detect the presence of multiple, genetically-different parasites in a single infection (later referred to as complexity of infection or COI). Whole genome sequencing studies have the potential to circumvent these limitations but i) are prohibitively expensive for analyzing hundreds of samples and ii) are hindered by the presence of the patient DNA and require processing the samples before sequencing (e.g., using CF11 columns [13] or sequence capture [14]).

We describe here a novel genotyping assay that provides sequence information from over 100 selected polymorphic loci and allows robust assessments of *P*. *vivax* population genetic diversity and COI. This technique enables high-throughput genotyping of parasite DNA without being hampered by human contamination (as *P*. *vivax* DNA is specifically amplified), provides a quantitative assessment of the proportion of each allele at the targeted SNPs, and can be easily customized to any parasite population or research question (as different markers can be investigated by simply changing the PCR primers). We demonstrate the applicability of this approach by analyzing close to 500 *P*. *vivax* infections collected throughout Cambodia. Our analyses show that the data generated enable the characterization of genetic diversity and population stratification in Cambodian *P*. *vivax*, and the assessment of COI in this region, as well as its main determinants.

## Materials and Methods

### Ethics Statement

This study was conducted in accordance to the Institutional Review Board (IRB) National Ethics Committee for Health Research of Cambodia (IRB 038NECHR) and the Cleveland Clinic IRB (IRB 12–936). All patients provided written informed consent for the collection of blood samples, with the understanding that samples would be de-identified before processing.

### Samples

**Fig 1:**
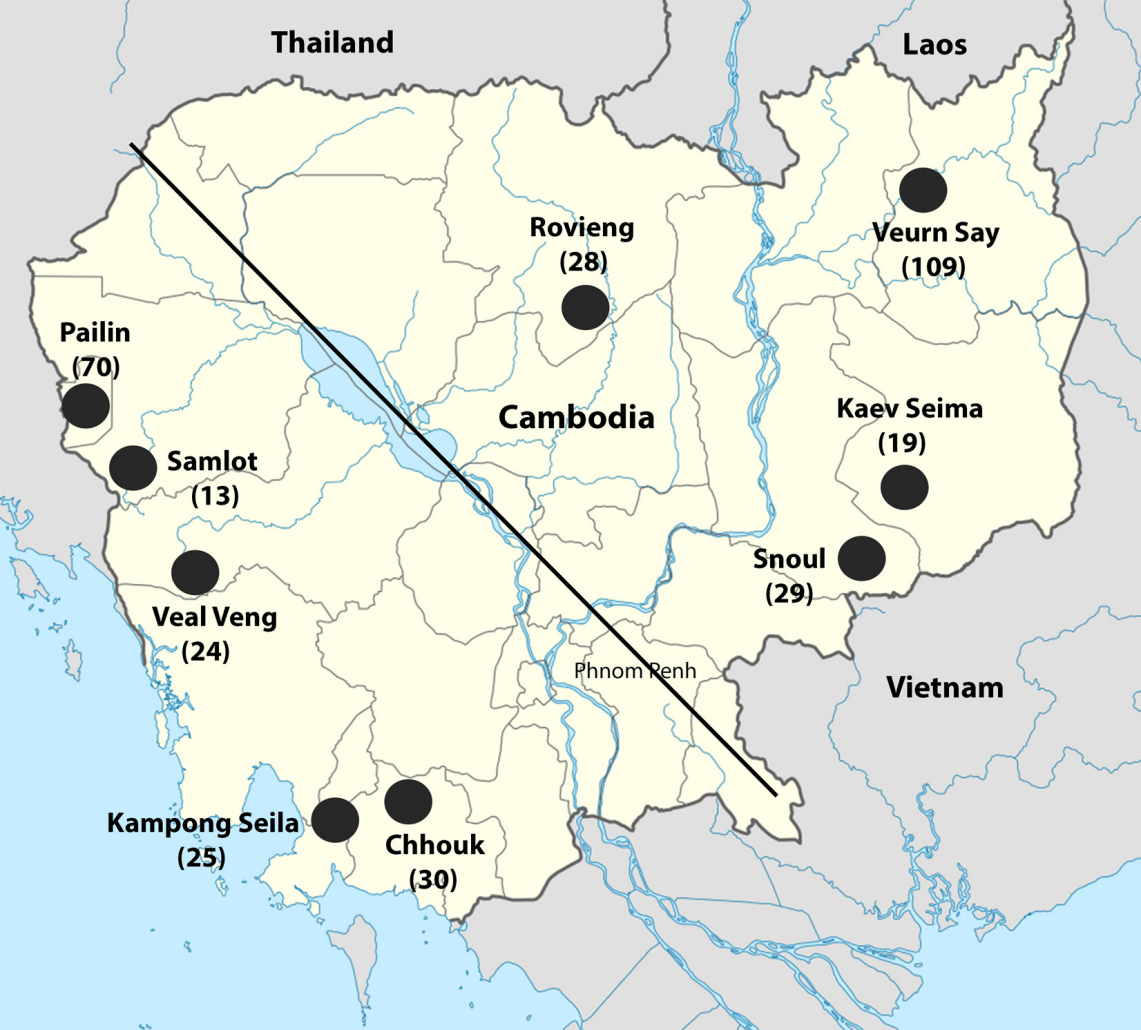
Map showing the approximate location of the recruitment sites in Cambodia. The diagonal line represents a no/low transmission zone and is used in our analyses to separate sites between western and eastern Cambodia. Numbers in parentheses indicate the number of samples included in the final analyses for each location.

We collected blood samples from 401 *P*. *vivax* symptomatic patients from nine locations across Cambodia (Fig 1) at three time points (in 2004, 2011 and 2013) and 88 asymptomatic patient samples from one district at two time points (2012 and 2013). Note that different individuals were recruited at each time (i.e., no repeated sampling of the same individuals over time). Demographic and clinical data on the samples analyzed are provided in S1 Table.

For each patient, we collected blood spots from finger prick or 5 mL of fresh blood in EDTA vacutainers and extracted DNA using a Qiagen Blood Mini Kit following the manufacturer’s protocol.

### Amplification by Locus-Specific PCR and Library Preparation

**Fig 2:**
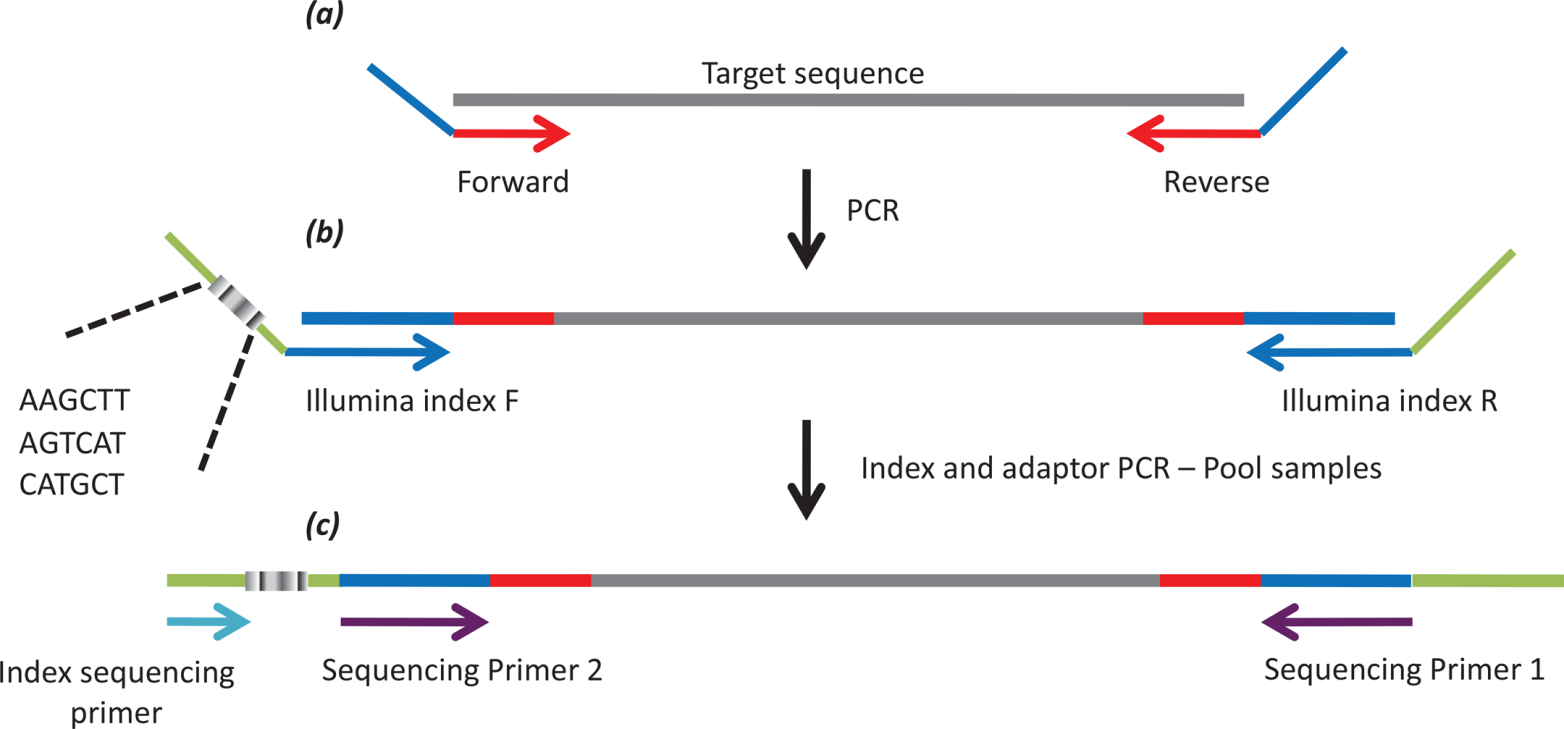
Overview of the genotyping assay. The grey bar represents the targeted DNA sequence containing a known SNP. A first PCR *(a)* amplifies 100–300 bp of target DNA sequence using locus-specific primers (in red) including a 5’end tail (in blue). Note that this first PCR is typically performed in multiplex with 8–10 primer pairs in the same amplification reaction. After pooling the amplified products from 13 independent multiplex PCRs, the molecules are amplified by a second PCR *(b)* targeting the 5’end tail. This PCR adds to each amplified molecule a oligonucleotide index specific to each sample (grey box) and the Illumina sequencing primers. The pools are then sequenced *(c)* to generate an index sequence and two overlapping reads.

We selected 128 SNPs among those for which both alleles were observed in at least two Cambodian infections that had been whole genome sequenced [15]. This condition was chosen to increase the chance that these SNPs were variable among Cambodian *P*. *vivax*. We used Primer3 v4.0 [16] to design primers targeting 100–300 bp around each selected SNP (S2 Table). Each primer included a modified 5’ tail to allow barcoding and incorporation of the Illumina sequencing primers by a second PCR (Fig 2). We pooled 10 mM of primers from eight to ten primer pairs (according to their melting temperature) and generated a total of 13 primer pools.

For symptomatic patient samples, we randomly assigned DNA into five 96-well plates and included duplicates for four samples as well as 25 water controls. For each plate, we conducted 13 individual PCR reactions (i.e., one primer pool per PCR reaction) to amplify all 128 SNPs using for each PCR, 1 μl of DNA (30–50 ng DNA template) in a 50 μl reaction consisting of 10 mM of each dNTP, 4 μl of 25 mM MgCl_2,_ 10 μl 5X GoTaq Flexi buffer, 10 μl of primer pool (containing 10 μM of each primer), and 1 unit of GoTaq DNA Polymerase. Reactions consisted of an initial denaturation of 3 min at 94°C followed by 30 cycles of 45 sec at 94°C, 45 sec at 56°C, and 45 sec at 72°C, and a final 3 min extension at 72°C. PCR amplification products were visualized on 1% agarose mini-gels stained with ethidium bromide.

We followed an identical procedure and randomly assigned asymptomatic patient samples into one 96-well plate containing three positive (DNA from symptomatic samples) and five negative (water) controls. For each plate we conducted 13 PCR reactions as described above to amplify all 128 SNPs, but using 45 cycles and Phusion High Fidelity Taq reagents.

We combined the 13 PCR products from each individual sample and purified them using a QIAquick 96 PCR Purification Kit. We then performed a second PCR to add to each PCR product i) Illumina adapters and ii) a 6-nucleotide sequence specific to each individual sample (Fig 2). PCR conditions were identical to those reported above except for the number of cycles (only 10 cycles were performed in this second PCR) and the primer concentration (1 μl of each primer at 10 μM). We then pooled together the barcoded PCR products from 96 samples into a single tube and purified them using a Zymo DNA Clean and Concentrator kit. To remove primer dimers, we excised and purified bands between 200 and 600 bp from a 2% agarose gel using a Zymoclean Gel Extraction kit. Finally, we verified the library quality and quantity by Agilent Bioanalyzer-2100 and sequenced each library (containing all PCR products from 96 samples) on an Illumina MiSeq to generate 11–16 million 250 bp paired-end reads.

### Sequence Analysis

We parsed all reads generated according to their respective 6-mer sequences and collapsed the paired-ends of each read into a single consensus sequence using PANDAseq [17]. We then mapped all consensus sequences to the *P*. *vivax* Salvador I strain reference genome sequence [18] using Bowtie2 v2.2.5 [19].

We eliminated from our analyses any targeted locus for which some of the amplified sequences mapped to more than one genome location, as this could indicate amplification of paralogous sequences. We also removed from further analyses loci that displayed more than two alleles at any given nucleotide position as this may also indicate amplification of paralogous sequences and, even if these alleles are genuine, would violate a common assumption of population genetic analyses (the infinite site model). Finally, we discarded loci for which different alleles show systematic differences in coverage (e.g., if across all infections, haplotype A was always represented by more reads than haplotype B) as this could indicate allelic dropout and poor amplification (e.g., the existence of polymorphisms at the primer sites). For symptomatic infections, we restricted our analyses to loci covered at ≥100 X in at least 100 samples. Individuals having less than 50 loci successfully genotyped based on the previous criteria were excluded. For asymptomatic infections, we included all individuals successfully genotyped (≥100 X) at least 1 SNP.

For each sample and each targeted SNP, we determined the coverage, and the frequencies of the reference and alternative alleles using samtools mpileup. Additionally, we determined the haplotypes at each locus for each sample by considering the combination of alleles present on the same read using the ‘MD’ string of the alignment files that summarizes all mismatch positions between the aligned reads and the reference genome. For both the targeted SNP genotypes and the locus haplotypes, we only considered alleles present in at least 10% of the reads of a given sample to avoid including PCR and sequencing errors.

### Complexity of Infection

We classified samples based on their detected clonality: samples that carried a single allele at all positions (i.e., the minor allele at any given nucleotide was never observed in >10% of the reads) were classified as monoclonal, while all other samples were considered polyclonal. For some analyses, we included, together with monoclonal infections, samples that displayed a single polymorphic site (i.e., only one nucleotide position showed two alleles) as for these infections we can reliably determine the combination of unlinked alleles carried by one parasite at all positions (i.e., the “phased” genotypes). We referred to these infections as biclonal.

We determined the combination of all major alleles across all initially targeted SNPs for the symptomatic infections (i.e., their “barcodes” [10]), to assess how informative these markers were at differentiating infections and calculated the mean number of nucleotide substitutions among them using MEGA v.6.0 [20]. For these analyses, we only considered monoclonal and biclonal infections.

We also determined the most likely number of clones present in each symptomatic infection using genotypes at the initially targeted SNPs and a maximum likelihood approach similar to the one implemented in COIL [21]: we estimated the population allele frequency at each initially targeted SNP using the mono- and biclonal infections and calculated, for each infection, the likelihood of the observed genotypes (i.e., either only the reference allele, only the alternative allele or both alleles at each SNP) when the infection contained 1 to 5 unrelated parasites. We thus estimated the most likely number of clones present in each sample. Note that the reference allele frequencies are very similar between the mono- and biclonal infections on one hand and the polyclonal infections on the other hand (S1 Fig) confirming that all these parasites belong to the same population.

We evaluated the correlations between different demographic and clinical parameters and COI using i) the assessment of monoclonality based on the presence of more than one allele at any sequenced locus and ii) the most likely number of clones in each infection. We tested the influence of geography (western vs. eastern Cambodia, using the low transmission zone as the boundary), age (<16 years of age vs. ≥16 years), sex (male vs. female), sampling years (2004, 2011, and 2013), glucose-6-phosphate dehydrogenase (G6PD) deficiency (according to reported World Health Organization guidelines), hemoglobin (Hb) genotype, and hemoglobin concentration (in g/dl) [22]. We also evaluated the effect of parasitemia (measured in parasites/μl) on COI and whether parasitemia itself was influenced by the other demographic and clinical parameters.

Finally, we tested whether the COI differed between symptomatic and asymptomatic infections. To account for the low genotyping success in asymptomatic samples, we randomly selected, for each asymptomatic sample, one symptomatic infection (from the same location and year) and used only the genotypes successfully typed in the asymptomatic sample to assess its clonality. We then compared the number of monoclonal infections in asymptomatic samples to the number observed in the randomly matched symptomatic samples. We repeated this procedure 1,000 times and calculated how often we observed more polyclonal infections in the symptomatic subsamples than in the asymptomatic infections.

### Population Stratification

We assessed population stratification in Cambodian *P*. *vivax* with the program STRUCTURE v2.3.4 [23] and the entire haplotype information at the targeted loci using all symptomatic infections determined to be either mono- or biclonal. To increase the discriminating power of our analyses and facilitate the interpretation of the results, we included genotypes at the same loci inferred from whole genome sequencing data generated from parasites collected in Thailand (*N* = 9) [24] and from non-Asian locations (*N* = 11) [15,25–27]. We assigned the parasites into *K* = 1 to *K* = 4 populations using 100,000 burn-ins, 1,000,000 Markov chain Monte Carlo (MCMC) steps and a model of correlated allele frequency with admixture. For each *K*, we compared the results from five independent runs to verify convergence to a similar solution and recorded the proportions of ancestry of each parasite.

We then tested whether the coefficients of ancestry of each parasite were significantly associated with geography (western vs. eastern Cambodia and between countries), age (child (<16 years of age) vs. adult (≥16 yrs.)), and sex (male vs. female) using *t*-tests. An ANOVA was used to evaluate differences among sampling years.

### Accession Numbers

The sequence data are available in NCBI SRA under the BioProject PRJNA295043

## Results

### High-Throughput Genotyping

**Fig 3:**
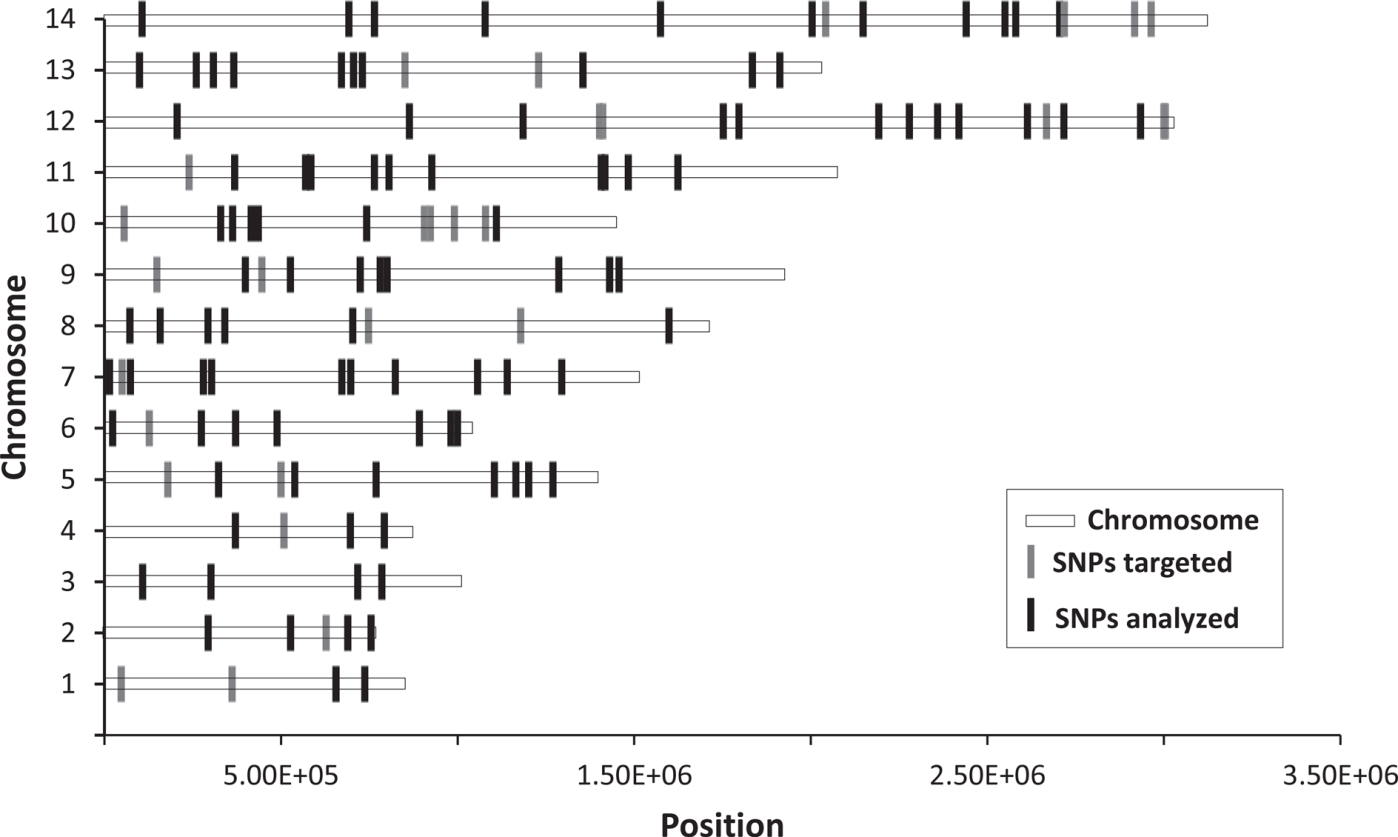
Genomic location of all targeted SNPs. The SNPs included in the final analyses are highlighted in black.

We developed a multiplex genotyping assay (Fig 2) that targeted 128 loci of 100–300 bp distributed throughout the *P*. *vivax* genome and covering all chromosomes (Fig 3). The loci were selected from a collection of SNPs found to be polymorphic among Cambodian *P*. *vivax* isolates that have been sequenced for their entire genome [15]. We applied this approach to analyze 489 *P*. *vivax*-infected individuals from Cambodia (401 symptomatic and 88 asymptomatic individuals). Out of the 128 primer pairs tested, one primer pair amplified DNA sequences that mapped to two different regions of the genome and was removed from the analyses. We also discarded one primer pair that amplified a low complexity locus causing misalignments in a homopolymer region. We additionally discarded six loci that displayed three alleles at some nucleotide positions (possibly indicating the amplification of unannotated paralogous loci) and 20 other loci that were not sequenced at 100 X in at least 100 symptomatic patient samples. The complete list of loci, their amplification primers and their inclusion in the final analysis is displayed in S2 Table. Overall, out of the 128 targeted loci, 100 were used in the final analysis. Four samples were analyzed in duplicate and showed very similar genotyping results (S2 Fig) suggesting that this approach did not generate many false positive calls.

Of the 401 initial symptomatic patient samples, 99 samples were excluded from our analyses since we could only reliably genotype them (i.e., sequenced by >100 reads at each locus) at less than 50 SNPs. Note that the genotyping success of symptomatic samples was not associated with parasitemia (*p* = 0.23), geographic origin (*p* = 0.21), or time of collection (*p* = 0.38). The samples included in the final analyses comprised infections from nine districts across Cambodia and three time points (Fig 1, S1 Table). The genotyping of asymptomatic patients was less successful and, out of the 88 initial samples, 43 were excluded from further analysis since they did not have 100 X read coverage at ≥1 SNP.

### Rigorous Identification of Complex Infections

*P*. *vivax* is haploid during the blood stage infection and monoclonal infections should therefore display a single allele at each nucleotide position. Using genotypes of the 100 SNPs that passed quality filters, 152 symptomatic patient samples, out of the 302 infections analyzed (50.3%), showed two alleles at, at least, one SNP, indicating the presence of two or more clones (i.e., polyclonal infections). For the remaining 150 samples (49.7%), the 100 SNPs all displayed a single allele and we were not able to rule out that these infections were monoclonal. Note that to limit the effect of amplification or sequencing errors, only alleles present in at least 10% of the reads were analyzed and therefore our analyses did not consider very minor clones.

Since our genotyping assay relied on high-throughput sequencing of 100–300 bp surrounding each targeted SNP, it enabled discovery and genotyping of additional DNA polymorphisms located in proximity to the targeted SNP position. In the 100 loci successfully analyzed in the 302 symptomatic samples, we identified a total of 274 SNPs. Using all 274 SNPs, only 25 infections (8%) were monoallelic at each nucleotide position (i.e., “monoclonal”) while 277 (92%) samples showed clear evidence of polyclonality. Of the latter, 60 infections (22%) displayed two alleles at a single locus. We referred to these samples as “biclonal” and included them with monoclonal infections in a subset of our analyses since they enable inferring a “phased” multi-locus haplotype for these samples (i.e., determining which alleles are carried by the same clone).

**Fig 4:**
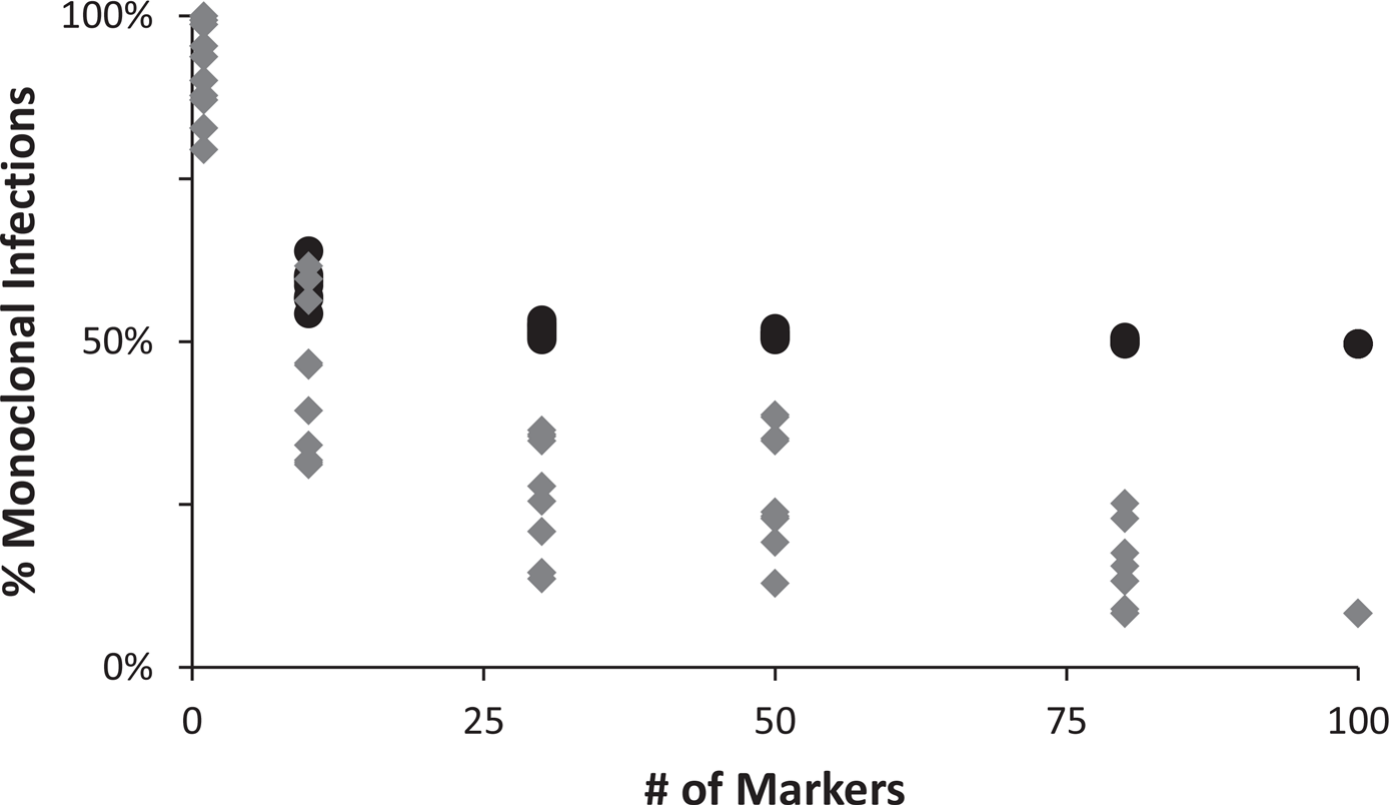
Proportion of monoclonal infected detected as a function of the number of markers genotyped. The black circles indicate the percentage of infections determined to be monoclonal using only single SNPs (i.e., the initially targeted SNPs) while the grey diamonds show the results using the entire locus sequences.

To assess how many genetic markers were necessary to rigorously identify COI, we randomly sub-sampled targeted SNPs and entire loci, and determined the proportion of infections deemed to be polyclonal using these data. Our results showed that single markers have limited power to identify COI and that more loci sequenced revealed more polyclonal infections, without apparent saturation (Fig 4).

We also estimated the most likely number of clones present in each infection using a maximum likelihood approach similar to the one implemented in COIL [21]. On average, each Cambodian infection contained 1.81 clones with 1.65% of the infections displaying more than three clones. Note that this analysis relied only on the 100 initially targeted SNPs and therefore underestimated the true COI (S3 Fig).

### Determinants of the Complexity of Infection in Cambodian *P*. *vivax*

We then investigated which parameters influenced COI in Cambodian *P*. *vivax* infections by i) comparing monoclonal and polyclonal infections (as determined by the genotypes at the 274 SNPs identified in the 100 loci sequenced), and ii) comparing infections with different numbers of clones (inferred statistically from the genotypes at the 100 unlinked SNPs). Using both data sets, we found that COI did not statistically differ between infections from western and eastern Cambodia (Table 1). Similarly, we found that COI was not significantly associated with parasitemia or the age or sex of the patient (Table 1). We also tested the association between various red blood cell phenotypes and COI but found no influence of hemoglobin level, hemoglobin genotype or G6PD deficiency (Table 1). Separating G6PD deficiency in mild and severe deficiencies, or restricting the analysis to male patients only (that are haploid for G6PD) did not change the results (*p*>0.14 and *p*>0.49, respectively). Note that, in this cohort, the parasitemia was not influenced by any of these parameters (Table 1).

**Table 1:** Association between clinical and demographic parameters and complexity of infection. The table shows the significance of the association (*p*-values) when comparing monoclonal to polyclonal infections (% monoclonal) or testing the correlation with the most likely numbers of clones of the infections (# clones). The association of the same parameters with parasitemia is also indicated.

	Parasitemia	% monoclonal	# clones
Sex	0.54	0.95	0.37
Age (< >16 years old)	0.85	0.98	0.79
Parasitemia	-----	0.54	0.66
G6PD (deficient vs. non)	0.32	0.88	0.09
Hemoglobin genotype	0.50	0.71	0.63
Hemoglobin count (g/dl)	-----	0.80	0.08
Geography (west vs. east)	0.14	0.28	0.18
Time of collection	0.76	0.03*	0.05*

*significant at *p*< = 0.05.

**Fig 5:**
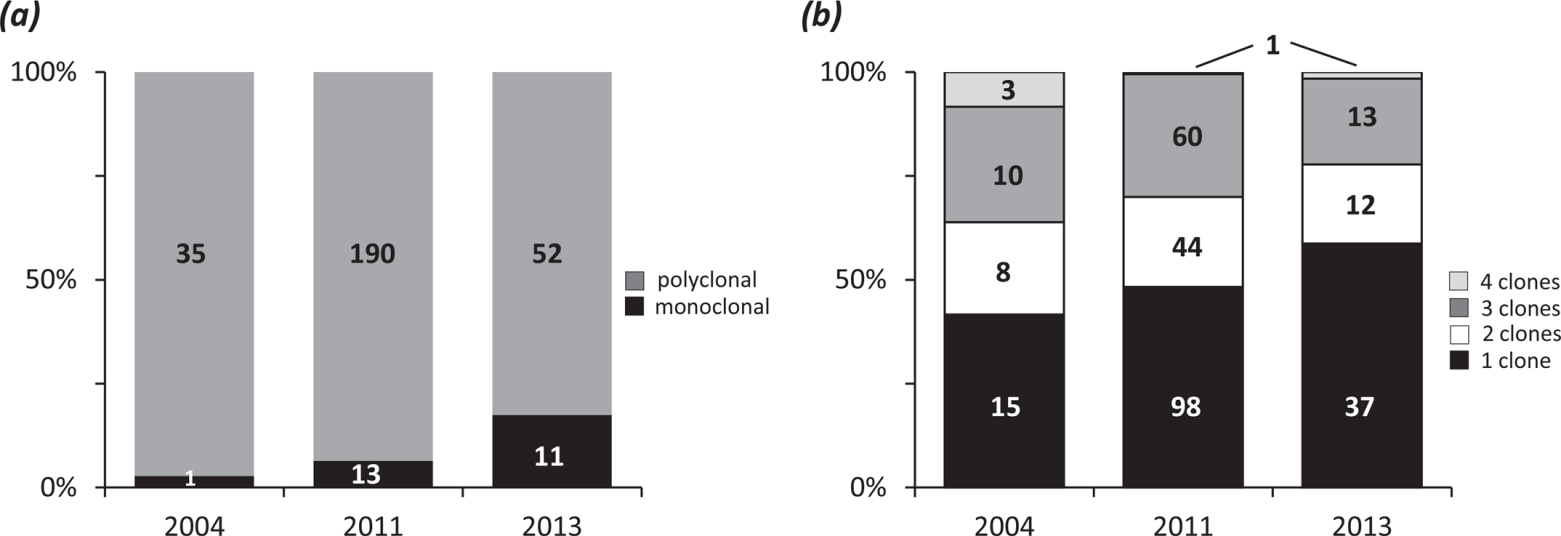
Complexity of infection according to time of collection. The infections are separated in monoclonal and polyclonal infections *(a)* or according to the most likely number of clones *(b)*. Numbers within the bars indicate sampling size for each category.

By contrast, we identified a significant association between COI and the time of collection (*p* = 0.03 and *p* = 0.05, respectively, for the comparison between monoclonal and polyclonal infections and the correlation between the year of collection and the most likely number of clones). The symptomatic infections collected in more recent years displayed significantly lower COI than older infections (Fig 5). The same pattern was observed by analyzing this association separately for each location (S4 Fig).

We then tested whether COI statistically differed between symptomatic and asymptomatic infections collected at the same location and the same time. To account for differences in genotyping success between symptomatic and asymptomatic samples, we randomly subsampled the symptomatic genotypes (see Materials and Methods for details) and showed that symptomatic infections were more often polyclonal than asymptomatic infections (*p* = 0.04).

### Genetic Diversity and Population Structure in Cambodian *P*. *vivax*

Population genetic analyses are complicated by the presence of multiple clones within an infection, which precludes reconstructing the genotype of each individual clone (i.e., the combination of alleles at different markers). We therefore limited our population genetic analyses to monoclonal (*N* = 25) and biclonal (*N* = 60) infections. Of these 85 parasites, 81 (95%) had a unique combination of alleles at the 100 initially targeted SNPs (i.e., a unique “barcode”), with a mean difference of 19.6 nucleotides between two infections. The infections caused by genetically undistinguishable parasites were always observed in the same location: two patients from Veurn Say were infected by parasites with the same genotypes at all 100 SNPs, as were a pair of patients from Rovieng and two pairs of patients from Pailin. These apparently similar infections at the same location could be caused by the same parasite infecting different individuals or by highly related parasites (e.g., recombinant clones). Overall, these findings indicated that most infections were caused by genetically very different parasites suggesting a high level of genetic diversity in the Cambodian *P*. *vivax* population.

We then assessed the population structure of Cambodian *P*. *vivax* using the entire DNA sequences of the 100 loci analyzed, treating them as multiallelic in the program STRUCTURE (using only genotypes at the 100 unlinked SNPs initially targeted yielded, qualitatively, identical results but with a lesser resolution, *R*^2^ = 0.83, *p*<2.2x10^-16^). To improve the power of our analyses, as well as facilitate the interpretation of the results, we included genotypes at the same loci from *P*. *vivax* parasites that have been sequenced for their entire genome [15,24–27].

**Fig 6:**
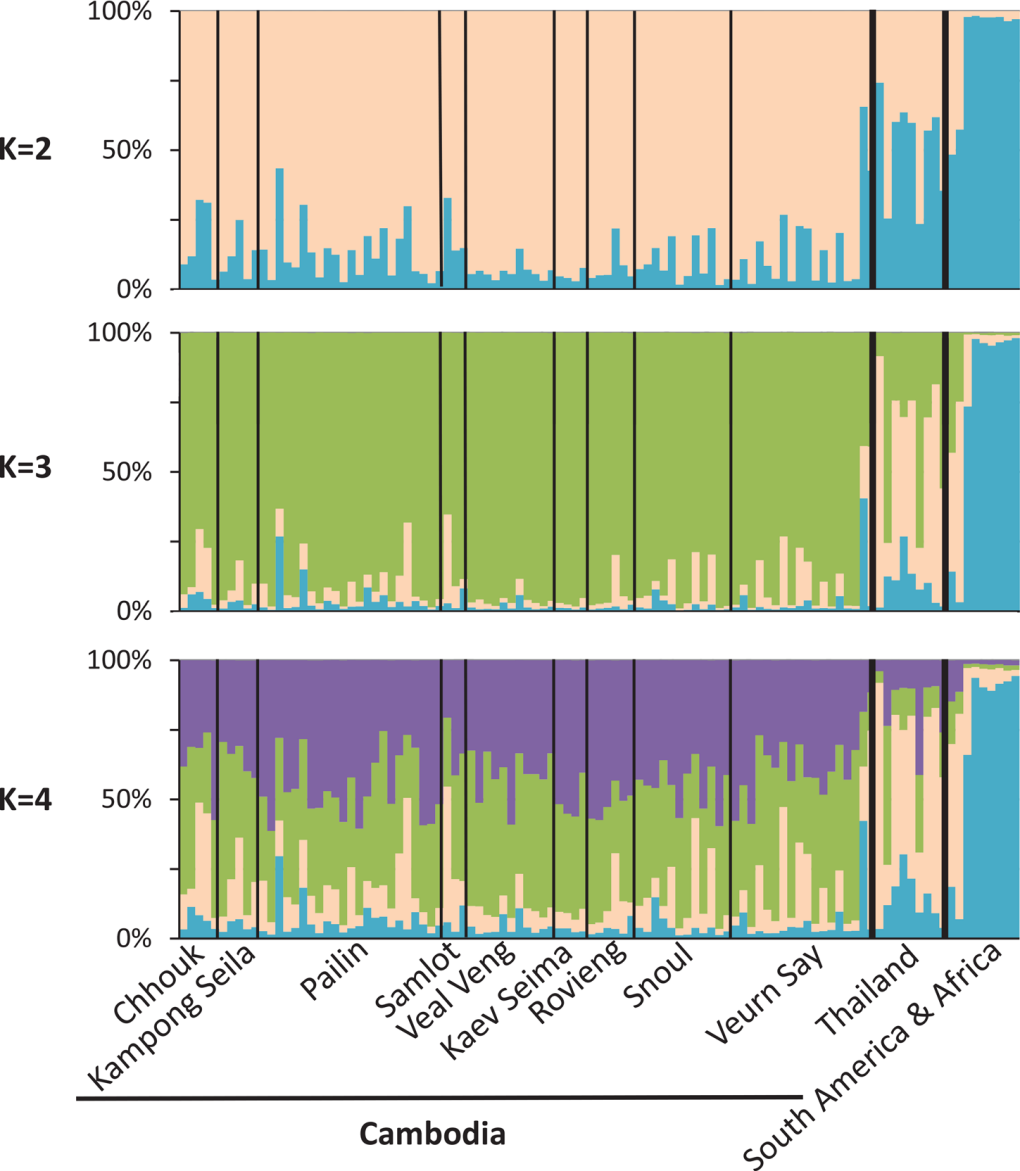
Population stratification analyses showing assignment of the parasites into *K* = 2, 3, and 4 populations based solely on their genotypes. Each vertical line displays the ancestry of one parasite (organized along x-axis) in each of the ancestral populations (represented by the different colors). Thin black lines separate districts in Cambodia. Individuals from Thailand and non-Southeast Asia are divided by thick vertical lines.

STRUCTURE assignments showed clear population separation between Southeast Asian (Cambodian and Thai) and South American and African parasites (*p*<2.15x10^-6^; Fig 6). Though Cambodian and Thai *P*. *vivax* showed ancestry from the same ancestral populations, they significantly differed in their coefficients of ancestry and appeared distinct (*p* = 0.0001; Fig 6). By contrast, we found no significant differences between parasites collected in western vs. eastern Cambodia (*p* = 0.09) or among sites (*p* = 0.09), suggesting that the Cambodian *P*. *vivax* population is little stratified geographically. Note that the parasite population also appeared to be genetically diverse in this analysis: the genomes did not fall into a single homogenous population, but rather were split into multiple populations (Fig 6). Additionally, we observed no association between the parasite genotypes and the age (*p* = 0.37), sex of the patient (*p* = 0.14), or with the sampling year (*p* = 0.79) (Table 1).

## Discussion

### A Quantitative and Customizable High-Throughput Genotyping Assay

Successful elimination of *P*. *vivax* will require monitoring how the parasite population responds to control measures to identify areas where elimination is less efficient due to logistical or biological reasons (e.g., high vector density or emergence of drug-resistant parasites). Several studies have used assessment of genetic diversity to measure *P*. *vivax* demographic parameters (e.g., see [28–32]). However, due to the general paucity of genetic markers in *P*. *vivax*, most past studies have relied on a reduced number of microsatellite markers or SNPs, or on DNA sequences at highly variable surface protein antigens. Unfortunately, these markers only provide a partial perspective on the parasite population and could be biased by variations in mutation rates or the role of natural selection (e.g., positive selection on the locus investigated could, for example, be interpreted as evidence of population expansion). Here we described the development and implementation of a cost-efficient and high-throughput genotyping approach that is easily customizable and enables rigorous characterization of COI, genetic diversity and population stratification in the population of interest.

The present assay targets over 100 SNPs distributed across the vivax genome, which minimizes potential artifacts in demographic inferences caused by the effect of natural selection driving the evolution of some markers. In addition, genotyping by high-throughput sequencing provides two additional key advantages. First, since 100–300 bp surrounding each targeted SNP are sequenced, the assay can identify additional DNA polymorphisms upstream or downstream of the targeted SNP position (for a total of 274 SNPs in our study) and provide haplotype data (i.e., multi-SNPs genotypes) that are much more informative than single SNPs (e.g., see Fig 4). Second, since more than 100 sequences are generated for each SNP and each sample, the genotyping is quantitative and enables high sensitivity and rigorous identification of alleles even if they are carried by only a minority of the clones (a major difference compared to traditional genotyping approaches that have limited power to identify rare clones [33]). Note that, by simply changing the amplification primers used, the assay can be easily modified to target any locus of interest and could, for example, include the recently developed “*P*. *vivax* SNP barcode” [10] to enable direct comparison across studies. This flexibility is also important as it enables optimizing the experimental design to specific research questions. We chose here to analyze a large number of markers and stringent coverage cutoffs to rigorously investigate COI (but with a relatively high rate of missing data). Alternatively one could reduce the number of markers or the depth of coverage to obtain a more complete dataset (but with lower power to detect minor clones).

### Complexity of Infection in Cambodian *P*. *vivax*

Overall, we observed a very high proportion of complex infections among Cambodian patients: 92% of all symptomatic infections showed clear evidence of having multiple clones. In 60 infections, we only detected a single variable position. This observation could possibly be explained by the presence of two closely related clones in the infection, similar to what has been described in *P*. *falciparum* [34,35]. However, most of the remaining 217 infections (72%) show numerous variable positions and likely represent instances of unrelated *P*. *vivax* clones simultaneous present in a given patient. This pervasive high COI contrasts with other studies that often show a large proportion of monoclonal infections (e.g., see [30–32]). While this discrepancy could be caused by lower parasite diversity or transmission rates compared to the situation in Cambodia, it is important to note that our estimates of COI are derived from sequencing numerous loci (instead of a few microsatellites or SNPs), which dramatically increases our power to differentiate clones and therefore to detect complex infections (Fig 4). In particular, our findings are consistent with previous studies that used next-generation sequencing approaches [15,36–38]. It is interesting to contrast these patterns of COI in *P*. *vivax* with studies of *P*. *falciparum*: for example, a recent study, including more than 200 Cambodian *P*. *falciparum* collected in the same areas as our *P*. *vivax* infections, showed that these samples were essentially clonal [39]. In this regard, our study confirms previous findings based on a small number of microsatellites that showed striking differences in COI between sympatric *P*. *falciparum* and *P*. *vivax* infections collected in western Cambodia [40]. This difference is COI could reflect the biological differences between these species and, in particular, the role of the hypnozoites that might contribute to the elevated COI in *P*. *vivax* by releasing previously dormant parasites in the blood stream upon a new infection [41]. Alternatively, this difference could be caused by a recent decrease in *P*. *falciparum* diversity due to extreme drug pressure in this region (though both parasite species are present in the same areas and are often treated indiscriminately).

We made use of the large number of samples investigated (*N* = 489) to test the influence of several patient and demographic parameters on COI. None of the patient data (age, sex, hemoglobin level or genotype, G6PD deficiency or parasitemia) were significantly associated with COI. In particular, higher levels of parasites in the blood were not statistically associated with polyclonal infections. Interestingly, we did detect modest but significant differences in COI between symptomatic and asymptomatic infections, with slightly more clones present in symptomatic infections. This observation will need to be confirmed by future studies but is consistent with the results of evolutionary models [42,43]. One could speculate that in *P*. *vivax* infections, which typically have a low parasitemia and multiple clones per infection, a higher number of clones could complicate the task of the immune system by presenting a more diverse range of surface antigens and lead to a more severe infection. In *P*. *falciparum*, increased COI has been associated with both lower and higher risk of malaria symptoms [44–47]. As more studies in both species become available, one will be able to rigorously address this interesting and clinically-important topic and test whether COI is associated with malaria symptoms and if the direction of this association is identical across *Plasmodium* species or in contrary, differs based on the specificities of each parasite (e.g., the differences in parasitemia and frequency of COI).

Some of the patient parameters investigated in our study had been previously reported to influence *P*. *vivax* infections [48–51]. For example, some hemoglobin variants [52,53] and G6PD genotypes [54,55] might confer some degree of protection against *P*. *vivax* infections. By contrast, our analyses showed that these parameters do not seem to influence the number of clones infecting a given patient. This apparent discrepancy between the influence of these parameters on *P*. *vivax* infection and on COI could indicate that the infection complexity does not arise from multiple independent infections but, instead, from either simultaneous infection by multiple clones (e.g., the mosquito might directly release multiple parasites in the blood) or by the relapse of dormant parasites from previous infections (see e.g., [56]).

Interestingly, the geographic origin of the patients did not correlate with COI despite the very different incidence of *P*. *vivax* in western and eastern provinces: the vast majority of reported *P*. *vivax* cases (80%) occurred in eastern Cambodia. By contrast, the year of collection was statistically associated with infection type: we observed that the proportion of monoclonal infections significantly increased in Cambodia from 2004 to 2013. This increase in monoclonal infection may reflect a general decline in the Cambodian *P*. *vivax* population, which may be indicative of the success of elimination programs [57] (e.g., see [58,59] for a similar pattern in *P*. *falciparum* in Senegal). Indeed, during this period, the number of malaria cases in Cambodia has been halved, dropping from 113,855 cases in 2004 down to 56,271 cases in 2014 (though a large component of this decrease can be attributed to a dramatic reduction in *P*. *falciparum* infections). This observation is encouraging for malaria control in Cambodia but analysis of additional samples collected more recently will be necessary to confirm this observation and to determine if this decrease is sustained over time.

### Genetic Diversity and Population Stratification of *P*. *vivax* in Cambodia

We observed a high level of genetic diversity in Cambodian *P*. *vivax* as illustrated by the large numbers of nucleotide differences differentiating clones, with, for example, an average of 19.6 differences, out of the 100 targeted SNPs, between two clones (and up to 40 differences). This high diversity is also illustrated by the results of our population structure analyses: most parasite genomes do not cluster homogeneously into one population, but instead display complex patterns of ancestry from multiple populations (Fig 6).

Interestingly, this extensive genetic diversity seemed to be distributed throughout Cambodia without clear demarcations: structure analysis failed to reveal any evidence of population stratification within Cambodia (Fig 6) despite the existence of a low transmission zone separating our western and eastern sampling locations (Fig 1). Similar results have been reported for *P*. *falciparum* populations [60]. While the 100 loci sequenced provide sufficient information to rigorously differentiate parasites from Southeast Asia and the rest of the world, and to detect significant differences between Cambodian *P*. *vivax* and (western) Thai *P*. *vivax*, it is possible that more genetic markers (or whole genome sequence data) would be necessary to identify subtle population differences within Cambodia. Regardless, two possible demographic scenarios could explain the lack of strong population stratification. First, it is possible that the current Cambodian *P*. *vivax* population derive from a single diverse ancestral population that was only recently split into multiple separated populations, and that these have yet to differentiate significantly. Alternatively, our observation could be consistent with a high level of gene flow between different Cambodian *P*. *vivax* populations, resulting in high level of admixture (which could explain the multiple ancestry patterns shown on Fig 6). However, differentiating these hypotheses would require extensive whole genome sequencing data that would enable rigorously quantifying gene flow, population stratification and the demographic histories of the parasite populations.

### Conclusion

Despite progress in malaria control in general, vivax malaria remains an important public health concern in many endemic countries. The lack of tools to assess the efficacy of on-going control efforts hampers the rapid implementation of alternative or complementary approaches in the field. We described here a novel genotyping assay that can interrogate over 100 SNPs across the *P*. *vivax* genome, in a rapid, high-throughput and cost-efficient manner and can be tailored to specific polymorphisms or loci of interest. Using this approach, we were able to characterize geographical and temporal variations in *P*. *vivax* genetic diversity in Cambodia. Our analyses revealed that the proportion of monoclonal infections is increasing in Cambodia, which may be an indication of the success of the on-going control measures. However, our analyses also suggest that gene flow among Cambodian *P*. *vivax* is important, which could be problematic if antimalarial drug resistant alleles arise in one site as they could then inevitably spread throughout the region. Overall, this high-throughput genotyping technique adds new options to our existing toolkit for malaria control by providing a rigorous framework to assess how parasite populations respond to disease control strategies.

## Supporting Information

S1 Table Number of *(a)* symptomatic and *(b)* asymptomatic samples included in this study according to their geographical origin. (PDF)

S2 Table Primer information for multiplexing assay. **Bold** denote SNP positions used for our final analyses.
(PDF)

S1 Fig Comparison of the allele frequencies estimated in mono- and “biclonal” infections and polyclonal infections. Each point represents one of the initially targeted SNP and is displayed based on its average reference allele frequency (RAF) in monoclonal and biclonal infections (x-axis) and in polyclonal infections (y-axis).
(TIF)

S2 Fig Comparison of the genotyping results between duplicated samples. Each plot corresponds to a different infection, with monoclonal samples being shown in *(a)* and *(b)*, and polyclonal in *(c)* and *(d)*. Each diamond represents one SNP and is displayed according to its allele frequency in the duplicated samples.
(TIF)

S3 Fig Complexity of infection in our symptomatic patient samples. The infections are separated in monoclonal and polyclonal infections *(a)* and according to the most likely number of clones *(b)*. Numbers indicate sampling size for each category.
(TIF)

S4 Fig Complexity of infection across years in *(a)* Pailin and *(b)* Veurn Say. The proportion of monoclonal infections is shown in black and the polyclonal infections in grey. The figures indicate the actual number of samples in each category.
(TIF)
