# Supplementary material for: Complexity of Infection and Genetic Diversity in Cambodian Plasmodium vivax
Source: PLoS Negl Trop Dis. 2016 Mar 28;10(3):e0004526. doi: 10.1371/journal.pntd.0004526 (PMC4809505; doi:10.1371/journal.pntd.0004526)
Supplement: S1 Table — (PDF) [file pntd.0004526.s001.pdf]

Table S1. Number of *(a)* symptomatic and *(b)* asymptomatic samples included in this study according to their geographical origin.

(a)

		Analyzed (tested)		
Province	District	2004	2011	2013
Kampot	Chhouk		37 (24)	24 (6)
Kampong Som	Kampong Seila		28 (25)	
Pailin	Pailin	37 (22)	28 (21)	29 (27)
Preah Vihear	Rovieng		31 (28)	
Kratie	Snoul		30 (24)	5 (5)
Pursat	Veal Veng		29 (24)	
Ratanakiri	Veurn Say	22 (14)	28 (25)	29 (25)
Mondulkiri	Kaev Seima		22 (19)	
Battambang	Samlot		22 (13)	
	Total	***59 (36)***	***255 (203)***	***87 (63)***
		Overall=401(302)		

(b)

		Analyzed (tested)	
Province	District	2012	2013
Ratanakiri	Veurn Say	62 (32)	26 (13)
		Overall=88 (45)	
