# Supplementary material for: Complexity of Infection and Genetic Diversity in Cambodian Plasmodium vivax
Source: PLoS Negl Trop Dis. 2016 Mar 28;10(3):e0004526. doi: 10.1371/journal.pntd.0004526 (PMC4809505; doi:10.1371/journal.pntd.0004526)
Supplement: S2 Table — Bold denote SNP positions used for our final analyses. (PDF) [file pntd.0004526.s002.pdf]

Table S2. Primer Information for Multiplexing Assay. **Bold** denote SNP positions used for our final analyses.

Chrom	Start	End	Length	SNP	Forward Primer	Reverse Primer	Closest Gene	Distance	Reason Eliminated	Median Cov. [95% range]
1	361352	361474	123	361432	GGAGCAAAGTCCGCAAAA	GTATCACGTGCGCGCTTT	hypothetical protein	1369	Low GC area	
	47425	47545	121	47513	CCAATACAGCCAAAGCCAA	TTCAATGTTTTGAGCAGGTTG	PST-A protein	60	Paralogous	
	**655536**	**655704**	**169**	**655621**	**CCGCGTCCTCAATGAGTT**	**TTCCTGAAGGACAAGCGG**	**DNA repair protein rhp54**	**0**		**769 [132-3290]**
	**737038**	**737218**	**181**	**737143**	**GCGGAGTGCATCCAAAAG**	**CCTCATCATCGGACCCAC**	**mannose-6-phosphate isomerase**	**0**		**567 [0-1948]**
2	**293428**	**293555**	**128**	**293524**	**GCTCCTCTTTCAGTTGGCA**	**GGACCACATGCACCACG**	**DNA repair exonuclease**	**0**		**2153 [202-7853]**
	**526973**	**527095**	**123**	**527036**	**AAAATTATGGGTCGCGCA**	**TTGGACACTGCTCTCGTTCT**	**hypothetical protein**	**1851**		**1002 [0-6495]**
	627509	627663	155	627583	CCCGAGTTCCCGTACAAA	CAATTTTGCGAATTGCCC	hypothetical protein	0	Paralogous	
	**688890**	**689086**	**197**	**689038**	**TGATGAATTCCGCCAAAAA**	**CCTCCCCACTTGGGAAAC**	**hypothetical protein**	**0**		**0 [0-531]**
	**754422**	**754528**	**107**	**754481**	**AGGACGAAGTTGTAGGTTTCG**	**GGTGTCGCACATTATTCCG**	**variable surface protein Vir22/23-related**	**0**		**1283 [198-5226]**
3	**107942**	**108060**	**119**	**107993**	**CCCACAAAGTTATGCGAGC**	**GCGTCAACACGGGTTTGT**	**hypothetical protein**	**115**		**1550 [292-7181]**
	**301609**	**301742**	**134**	**301681**	**CGTCGTGAAAGTCCGCTT**	**ATCAGGCAGCTCTGTGGG**	**hypothetical protein**	**0**		**454 [0-3947]**
	**717058**	**717191**	**134**	**717122**	**AGGAGGGCCAAACGAAAC**	**GCCTCTCTCTGCACCGTC**	**hypothetical protein**	**0**		**763 [0-3704]**
	**785420**	**785618**	**199**	**785501**	**CCGCTCCCATCAGAAAGA**	**GCCAGAACATCCCCATCA**	**hypothetical protein**	**0**		**248 [0-774]**
4	**370747**	**370944**	**198**	**370876**	**CGCACGGATAATAAGCGG**	**ACTATCCCCCTCATCGCC**	**hypothetical protein**	**0**		**1220 [224-4638]**
	508241	508407	167	508314	GCTGCACTTTGAGGACGTT	GGGGGAGAGCGAAAAGAA	transmission-blocking target antigen Pfs230	0	<100 inds. covered	
	**696217**	**696343**	**127**	**696267**	**CGGAGATGACCCACAAGG**	**ACTGACTTGGCGTCTGGG**	**hexose transporter**	**0**		**587 [0-3391]**
	**792410**	**792566**	**157**	**792480**	**AGAAGAAGCCGCTGCTGA**	**GACGGTTGCATCACCAAA**	**hypothetical protein**	**0**		**802 [148-3945]**
5	**1103916**	**1104057**	**142**	**1104008**	**AAGAGGCACCTGCACAGC**	**ACTGGTGGAGGGCATTCA**	**hypothetical protein**	**0**		**351 [0-1964]**
	**1164807**	**1164915**	**109**	**1164846**	**CCATTGGGTGGTTGATGG**	**ATCGAACGGAACAGCCAC**	**hypothetical protein**	**2670**		**2430 [291-7875]**
	**1201219**	**1201418**	**200**	**1201371**	**ACTCGAGCCAGTGCCCTT**	**TGCTCGACGCTTTCACAA**	**helicase**	**0**		**0 [0-571]**
	**1269930**	**1270054**	**125**	**1269980**	**CCAATTTTCCCACTGTTCATC**	**GGGGAGATTGGGTAAAATCC**	**hypothetical protein**	**205**		**365 [0-1707]**
	179439	179617	179	179561	CGACAAGTGGTGGAGCAA	CATGCGTTCCTTTGGAGG	hypothetical protein	0	<100 inds. covered	
	**323132**	**323307**	**176**	**323176**	**CCAAAGCGGGAAAGGAAT**	**TTTCGCTATCGAGGGTGC**	**phosphatidylinositol 3- and 4-kinase**	**0**		**509 [116-2642]**
	499883	500049	167	499928	CCGATCGGAGTAGGAATGG	CAGGAATGGCTCCCCTTT	hypothetical protein	0	<100 inds. covered	
	**538652**	**538811**	**160**	**538751**	**AACCGGTGCACATATCCG**	**CAGGTCATTTTGGGCGTT**	**40S ribosomal protein S19s**	**0**		**3769 [817-7788]**
	**769054**	**769158**	**105**	**769107**	**TGGGAAATCAATGCTGTGC**	**CTCAACACACTTGGGGGC**	**Pv-fam-a**	**0**		**101 [0-1194]**
6	126785	126886	102	126837	ACGCGTCGAACAGTAGGC	AGTGGGTCTCGCACAACC	hypothetical protein	2882	Paralogous	
	**23060**	**23171**	**112**	**23136**	**ATTATTGCAGCCCCCACA**	**CGCCTTCTACACTGCCTG**	**hypothetical protein**	**355**		**0 [0-6671]**
	**274284**	**274480**	**197**	**274421**	**ATGCCCAGGGAGTGAATG**	**TGCCAGCAGAATGAGGTG**	**hypothetical protein**	**0**		**430 [0-2173]**
	**371371**	**371496**	**126**	**371442**	**CCTAGTTGAACGCGTGGC**	**TCGTGGAGCTGCAAAAGA**	**hypothetical protein**	**815**		**1322 [0-5838]**
	**488465**	**488638**	**174**	**488587**	**ACTTGGTCACGCTGCACA**	**TCGAGCTCAGCTTTGATGG**	**hypothetical protein**	**0**		**351 [0-1274]**
	**891574**	**891761**	**188**	**891710**	**TCCACGTGAACTCGCTGA**	**GAACCCCTCAAATTGGGC**	**hypothetical protein**	**0**		**412 [0-1988]**

	**980802**	**980992**	**191**	**980923**	**CACGTAGAACAAACTGGCGA**	**TTGGTCAGCGTGGAGATG**	**hypothetical protein**	**0**		**690 [121-2770]**
	**999481**	**999608**	**128**	**999554**	**CCCTCGTGCTGTTCGACT**	**GCTCAGCTTCACCAGGGA**	**dynein heavy chain**	**0**		**723 [0-5300]**
7	**1056525**	**1056629**	**105**	**1056571**	**CCTTTGGACCCACCACAG**	**CAACGGACGACCTGTTCC**	**RBP1**	**0**		**357 [0-2394]**
	**1140421**	**1140615**	**195**	**1140487**	**AAGGCGATGCTTCTGCTG**	**GCTAACCACCCCCATGAA**	**hypothetical protein**	**0**		**375 [0-2327]**
	**1294794**	**1294993**	**200**	**1294884**	**AGAATCCAAACGGGGACC**	**GCGGGCTGAGAGACTTGT**	**hypothetical protein**	**0**		**525 [112-1708]**
	**14237**	**14387**	**151**	**14335**	**GCTGTGTTCCATTTGCTTCA**	**TTTATTCCACCAAAATAGCGA**	**hypothetical protein**	**4032**		**682 [0-2460]**
	**280227**	**280339**	**113**	**280259**	**GGGCAATGCTCAGTGGAC**	**GTTTGGGACCCCATCCTC**	**hypothetical protein**	**0**		**3691 [729-7928]**
	**303320**	**303502**	**183**	**303395**	**CGAAGAAATTTACACCTCCCT**	**TGATCGTTTACATTTTCGAGG**	**GRP 78**	**0**		**161 [0-1235]**
	49782	49905	124	49817	TAAAATTGCGCCCAAGGA	CCACGTTTCCCGTCTGAA	hypothetical protein	177	<100 inds. covered	
	**671952**	**672119**	**168**	**672072**	**CGCAAAATATGAGAAGAGCAA**	**ATTCCAGCTTGGATGGCA**	**hypothetical protein**	**0**		**445 [0-2363]**
	**697275**	**697401**	**127**	**697371**	**AAAACCACACGCGGAGAA**	**GGGAGGTGGCAAGGTTGT**	**fumarate hydratase**	**2809**		**2421 [473-7560]**
	**73308**	**73487**	**180**	**73440**	**CTCCGTTCAGGAAAACGC**	**CGCTTCAACAACAAAAGCG**	**hypothetical protein**	**0**		**329 [0-2385]**
	**823310**	**823479**	**170**	**823425**	**ACATGGCTGGGAATGCTC**	**GTGGAAAACACCAACGGG**	**delta tubulin**	**704**		**2566 [575-7661]**
8	1178508	1178632	125	1178586	ACGCAAAAGCGCTCGTAT	CGCGAATGCATACGTAAAA	CLAG	194	Paralogous	
	**157548**	**157676**	**129**	**157614**	**GACACAGCGGAAGCCCTA**	**TGTTGCGGACTCCCTCTT**	**hypothetical protein**	**0**		**860 [166-3511]**
	**1598410**	**1598606**	**197**	**1598514**	**CGCTGACCAACTACGGCT**	**CTGCACAGCTTCCTTCAGC**	**hypothetical protein**	**0**		**692 [124-2379]**
	**293010**	**293178**	**169**	**293076**	**AGCATGCGCGTGTTGTTA**	**CTCACTGGGTGTGCCTCC**	**hypothetical protein**	**185**		**787 [110-4060]**
	**340616**	**340712**	**97**	**340672**	**AGGAGGGGGATTCAAAGG**	**CCCCCTCTCCCTTTTAGC**	**hypothetical protein**	**844**		**256 [0-1239]**
	**702929**	**703037**	**109**	**702981**	**CCAGGTGGTCATTCAGCA**	**GAGGACAACCCGGAAGGT**	**hypothetical protein**	**0**		**1328 [226-6435]**
	**72037**	**72193**	**157**	**72119**	**GCTGAAGGGAATGTTTCTCC**	**CCTTGCAGGCATTGTCAG**	**PFC0600w**	**0**		**189 [0-1246]**
	747572	747697	126	747638	CGCTTCTTGGTTGGCACT	CCGGCTCACCCCTTCTTA	N-ethylmaleimide sensitive fusion protein	0	<100 X coverage	
9	**1286337**	**1286447**	**111**	**1286388**	**GTTTCCGCCCCTTTGACT**	**AATAGCAGGAACGGCGTG**	**30S ribosomal protein S15**	**0**		**774 [137-3958]**
	**1430373**	**1430519**	**147**	**1430436**	**AGCGATTTGGAGGCTGTG**	**GTGGAAAAATTGCCCCCT**	**hypothetical protein**	**0**		**2937 [0-7837]**
	1457084	1457226	143	1457147	GCTCTGCTTCGTATGCTGG	GGGGGATGAACAAGCTCTC	dynein light chain type 2	0	<100 X coverage	
	**148205**	**148401**	**197**	**148307**	**TGCTCGTCTACCTGGGGA**	**CACCTTCCTGCTCTTCGC**	**hypothetical protein**	**0**		**2083 [455-6548]**
	**398650**	**398826**	**177**	**398781**	**TGGCGTCTTCCTCCTCTG**	**AACTGCTGCTCATTCGTCG**	**WD domain, G-beta repeat domain**	**0**		**270 [0-2476]**
	445642	445835	194	445710	TGGAACGTCGAAACGGAT	AAAATGCGCTGACGTGTG	hypothetical protein	0	<100 inds. covered	
	**526505**	**526619**	**115**	**526557**	**GCCCACGCAGGTACACTC**	**TCGGCGTTTTGTCTCTCC**	**dynein heavy chain**	**0**		**433 [0-2511]**
	**724225**	**724324**	**100**	**724256**	**CCGTGTTCCGTTCAGCTT**	**TGCTACACCCTTCTGCCC**	**hypothetical protein**	**1101**		**1805 [273-7898]**
	**780417**	**780597**	**181**	**780482**	**GGAGACAGCGTCACGGAA**	**TCTTGAACACATTGATGGGG**	**hypothetical protein**	**0**		**906 [200-3702]**
	**800235**	**800412**	**178**	**800284**	**GGAACAACGAGTGGTCCG**	**TTCAGCTCTGCGTTGGTG**	**Adenylate and Guanylate cyclase domain**	**0**		**199 [0-1379]**
10	1078888	1079047	160	1079004	GCACCGTCTGCCATCATT	ACAATCAGGGTGGCCAAA	XAP-5 DNA binding protein	2867	Paralogous	
	**1109668**	**1109797**	**130**	**1109736**	**CCCCCTGACGTAAAAGAACA**	**TGTCACGCCCATCAGTCT**	**hypothetical protein**	**0**		**1038 [6-4241]**
	**328962**	**329134**	**173**	**329085**	**AATGCACATCCCAATGGC**	**GAGGATGCTGCGGATGAT**	**mdr1**	**0**		**833 [181-3082]**
	**362742**	**362932**	**191**	**362870**	**CATCAACTTCCCGGCGTA**	**AAGGACAAAAGAGAAAGACGC**	**hypothetical protein**	**0**		**1692 [323-6884]**

	**415472**	**415600**	**129**	**415514**	**TCGTTGTTCCCCTGTGCT**	**GCACATCCAGGAGAGCGT**	**hypothetical protein**	**0**		**358 [0-3215]**
	**435461**	**435618**	**158**	**435533**	**AAGTCGTGGAAAATTACCCTG**	**AAGCTGCACCTGAACTTGC**	**asparagine-tRNA ligase**	**0**		**144 [0-946]**
	55543	55741	199	55617	CAGCGTCCAAGTGGGAAA	GGGAAGGGAAGCCAAAAT	topoisomerase I	0	<100 X coverage	
	**742509**	**742643**	**135**	**742568**	**CTTGCCTCGCAGAAGGAG**	**GCGGCGGCTAAATGAGTA**	**hypothetical protein**	**0**		**430 [0-2317]**
	905752	905951	200	905853	CGCGTTACCAGTCCGTTT	CGCTTTCGTCTCCTCCTTC	ATP-dependent RNA helicase	0	<100 inds. covered	
	922743	922867	125	922798	GAAAGCGCCATTTGAAGC	GCTTGAGCCTGTTTGCGT	hypothetical protein	816	<100 inds. covered	
	990626	990818	193	990752	GGAGGACCACTCTCCAAGG	GCAGACGGCCATGATTTC	hypothetical protein	558	<100 inds. covered	
11	**1406505**	**1406672**	**168**	**1406626**	**AGGAGGAGCAGCAGCAGA**	**CGCTGGAAAATTGCTCGT**	**oxidoreductase, aldo/keto reductase domain**	**0**		**784 [0-4040]**
	**1417191**	**1417307**	**117**	**1417248**	**TGGATTAGCTGCAACGGG**	**CGCACATAGGAGGGAGGA**	**hypothetical protein**	**0**		**5174 [1109-7938]**
	**1483164**	**1483363**	**200**	**1483242**	**AAAAACGTTGTGGAAGTCGG**	**TCCTTGTCCAGCTCGCTT**	**hypothetical protein**	**0**		**1576 [392-4936]**
	**1623846**	**1623946**	**101**	**1623889**	**CGCTTTCGAACTTCTCCTTC**	**CGTGGAGGGAGCAAAAGA**	**hypothetical protein**	**0**		**0 [0-1242]**
	239465	239580	116	239520	TCTGCATTCTCCCCTTCG	TTGTAGGCCCCAAAATGC	hypothetical protein	0	Paralogous	
	**368720**	**368918**	**199**	**368766**	**CCATTTGTTCGTGAAACTCG**	**GATTGCGCAAGTGCTGTG**	**SET domain containing protein**	**0**		**0 [0-326]**
	**569502**	**569699**	**198**	**569629**	**GTGCCTGTCGATGAAGGG**	**CCCCCAGACTCATTCACCT**	**transportin**	**0**		**144 [0-1320]**
	**584709**	**584811**	**103**	**584756**	**GCAAGTGCATGGGCGTAT**	**TGACGGGTTAGCGGGTTA**	**hypothetical protein**	**0**		**3544 [0-7828]**
	**764284**	**764461**	**178**	**764358**	**CGGAGCTGTCTTACATGAGC**	**CTGACCGTTTCGTTTGGAA**	**hypothetical protein**	**0**		**0 [0-366]**
	**805623**	**805807**	**185**	**805674**	**CGGTACACGTTTTCGCCT**	**GCCACTGGGATGAGCAAC**	**syntaxin binding protein**	**8199**		**697 [123-2361]**
	**926860**	**927035**	**176**	**926923**	**TTCCTGCAAGTTCTCCGC**	**GGAGGCTCTCCTCCACCT**	**hypothetical protein**	**0**		**408 [0-1721]**
12	**1185138**	**1185332**	**195**	**1185180**	**GGGGACAAAACGACCCTC**	**AGGCACTCTCCACAGCGA**	**hypothetical protein**	**0**		**164 [0-1570]**
	1401793	1401922	130	1401871	CATGCGTGTGAATGCTGC	GTGCCACGTGTGGGTACA	hypothetical protein	315	<100 X coverage	
	1410878	1411072	195	1410920	TCCCAAATCTGGTGGGAA	GGGCCGGCTAATTTTTCT	hypothetical protein	0	<100 inds. covered	
	**1752169**	**1752350**	**182**	**1752233**	**ACGTGGCGGATAAGAGGG**	**GCCACTACAGCGCAGCTT**	**20S proteasome beta 4 subunit**	**0**		**168 [0-778]**
	**1796443**	**1796554**	**112**	**1796509**	**CAGTTCGGACAGCGACAA**	**CTCCATATGCCCATTCGC**	**hypothetical protein**	**0**		**1034 [139-5381]**
	**205594**	**205775**	**182**	**205690**	**TCCACCCCTGTACGTGCT**	**GCATGCAACTCACCATGC**	**hypothetical protein**	**0**		**358 [0-2353]**
	**2192449**	**2192616**	**168**	**2192552**	**TTCCATTCCCCTTCAGCA**	**TTGAGGAAGTCCCTGTGTGT**	**hypothetical protein**	**2496**		**391 [0-1726]**
	**2279092**	**2279273**	**182**	**2279221**	**TCGTCTTCACTCACCTGCC**	**GACCACCGGAAAAACACG**	**hypothetical protein**	**0**		**1490 [335-5656]**
	**2358955**	**2359069**	**115**	**2359028**	**CCCTCGTGTTTGTCACTGG**	**AACACCTTTGCTAACCGCC**	**hypothetical protein**	**0**		**1375 [168-7037]**
	**2419277**	**2419476**	**200**	**2419371**	**AAATGTCCGCCTCACTCG**	**TAAGCAGGCGTCTCCCAC**	**hypothetical protein**	**0**		**1538 [241-5207]**
	**2613332**	**2613452**	**121**	**2613369**	**CGATTGCAGCTACACGGA**	**CGCATAGTCTAAGCCCACG**	**hypothetical protein**	**0**		**2255 [534-7906]**
	2667435	2667596	162	2667491	CCGCCAAAGAGAGAGTCG	TAGCTCGTTCACCTGGGC	serine/threonine protein kinase	0	<100 inds. covered	
	**2716555**	**2716680**	**126**	**2716622**	**CAGCCAAAATGGCGAACT**	**GGACAACTTCCACGTCGC**	**methionine-tRNA ligase**	**0**		**2318 [475-7859]**
	**2933845**	**2933985**	**141**	**2933890**	**AGTAATGCTTGTCAGTGGGC**	**TTGTTCGCACGCAAGAAA**	**hypothetical protein**	**3776**		**433 [0-2059]**
	3003711	3003890	180	3003775	GATGACGGTTTTTCTTCCCA	GAAAATGTCACCAAGAAGGGA	hypothetical protein	0	Double mapped	
	**863242**	**863402**	**161**	**863346**	**TGTTTGGCGGTGTCTCCT**	**ATCGCCAGTTTGGTCCAG**	**hypothetical protein**	**0**		**345 [0-3287]**

13	1229435	1229556	122	1229481	CCAACCGGGGGAAGTAAC	GCAGGTTCGGTTTTCCCT	hypothetical protein	0	<100 inds. covered	
	1354865	1355061	197	1354999	GTCAGCAGCATGCACAGG	CGCGGTTATCCAAACCAA	hypothetical protein	0		317 [0-1101]
	1834633	1834806	174	1834684	TTGAGGAGACGTGCAAGAAA	GGTCCAGGTTTTCCTCCG	cysteine repeat modular protein	0		858 [0-3459]
	1912275	1912461	187	1912361	GAGGAGTTGTCGCGCTGT	GATGCCACATCCTCCACC	hypothetical protein	0		763 [184-3535]
	259820	259990	171	259864	TGCGCACTTTCCGTATCA	CAGCGATGAGGAGGAGGA	hypothetical protein	0		385 [0-1591]
	308408	308605	198	308456	TGGAGAAGCCAACCCTCC	TGCGAAATGGAGAAACGTC	hypothetical protein	0		145 [0-1111]
	366198	366303	106	366254	ACTTGGCTGTTGGCAAGC	AGCAGATGCGGAAGATGC	hypothetical protein	0		317 [0-2736]
	670820	671008	189	670891	AAGGGCTTCAAGAGCGGT	TTGCTCCCCAGATTGCTC	hypothetical protein	0		493 [0-2448]
	705334	705527	194	705424	AACGTGGGTGTTTCCCCT	TCCACCTCGCAATCTGTG	hypothetical protein	0		340 [0-1322]
	730306	730406	101	730336	GCGAAATGATGAAGAGCGA	CCCCAGGTTCATGCTGAC	lysine-tRNA ligase	0		280 [0-3664]
	850825	851000	176	850957	CGCTCCTACCGCTGCTAC	CTTTTCCACCCTTCCGGT	hypothetical protein	0	<100 inds. covered	
	99127	99251	125	99198	TTTTTCATTTCGTTACCCCC	TGCATGCAGGAAGGTGAA	hypothetical protein	88938		841 [0-4711]
14	106446	106575	130	106485	ACGAAAAACAAAGAGAAAGCC	TTGTTTGAAATGGCTGTTTG	RBP2	0		577 [0-1842]
	1077815	1077990	176	1077928	ACTGCAGGTCGAAGTGGC	TACTACGTGGAACGCGCA	hypothetical protein	0		4428 [962-7826]
	1574385	1574501	117	1574423	TCAAATTTCGGACCAGCC	GGGGCTCCAAATCAAAGG	hypothetical protein	338		1631 [326-6380]
	2003677	2003839	163	2003760	ACCTCTCCCCAGACGGAC	TACTTCCGGCACAGGCTC	hypothetical protein	0		219 [0-766]
	2042192	2042384	193	2042312	CGTGATGTGCGCGAAGTA	CGTCTCGGACGACTGCAT	hypothetical protein	817	<100 inds. covered	
	2148049	2148230	182	2148184	TTTTGGCATTCATCCCGT	GGGAAACCTCCAGAGGGA	hypothetical protein	0		0 [0-727]
	2440013	2440185	173	2440112	AGTCCCACGAAGAGCTGC	GACCTCGCTCGGAGCATA	hypothetical protein	0		143 [0-1010]
	2549815	2549929	115	2549860	AAGCGCCCTTGTTGTACG	GGCCATATTGCCAACACC	hypothetical protein	0		469 [0-3758]
	2580135	2580258	124	2580222	GGCCAGGCATGGCTACTA	GCCACTTTCCAACTCGTGA	hypothetical protein	0		247 [0-1065]
	2704477	2704623	147	2704545	AGAAGGGGGAGTTCGTCC	TGGCACTTGTGTGCTGGT	hypothetical protein	0	<100 inds. covered	
	2717974	2718077	104	2718016	ACAGGAGCATCGTCCGAG	CCGCAGGAAGGGAAGAAG	queuine tRNA ribosyltransferase	0		319 [0-1400]
	2916807	2917006	200	2916900	TTTGGTACGCCCCTCATC	GACCTTTCGGGAAATGTTCA	hypothetical protein	0	<100 inds. covered	
	2963777	2963972	196	2963845	CGGTCATCATTTGATCTTCGT	GCGCAAAAGAGATCTTACGAC	hypothetical protein	1673	<100 inds. covered	
	692050	692175	126	692128	ATAAACCGCACCACCACG	CACGTCAAACACGTAGGGC	tryptophanyl-tRNA synthetase	0		3480 [349-7850]
	764560	764728	169	764662	TCGATCGAGTCGCCTAAAA	GCCACTGCTCCGAATGTT	hypothetical protein	191		0 [0-595]
