# Supplementary figures and images for: Complexity of Infection and Genetic Diversity in Cambodian Plasmodium vivax
Source: PLoS Negl Trop Dis. 2016 Mar 28;10(3):e0004526. doi: 10.1371/journal.pntd.0004526 (PMC4809505; doi:10.1371/journal.pntd.0004526)

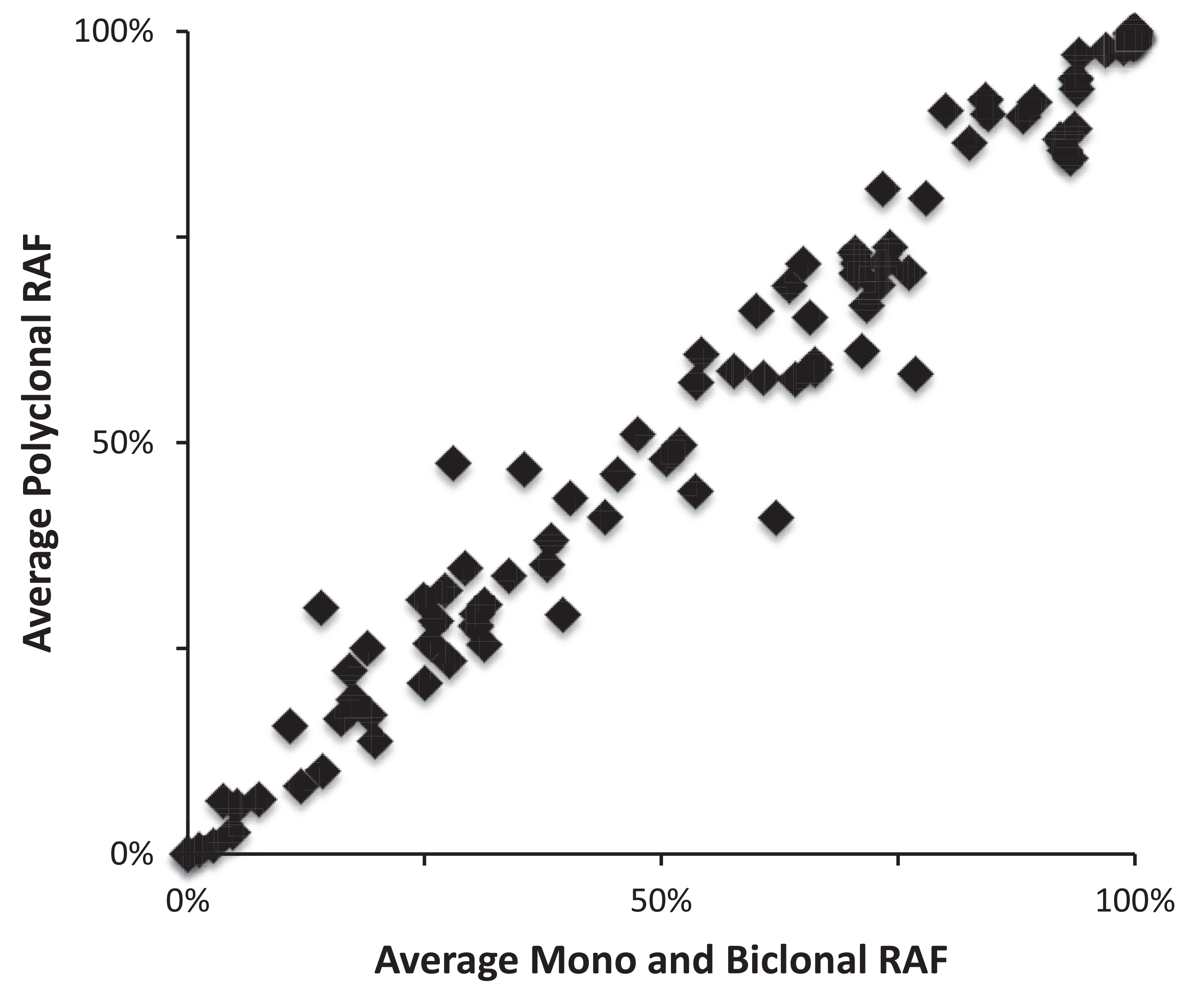

Supplement: S1 Fig — Each point represents one of the initially targeted SNP and is displayed based on its average reference allele frequency (RAF) in monoclonal and biclonal infections (x-axis) and in polyclonal infections (y-axis). (TIF) [file pntd.0004526.s003.tif]

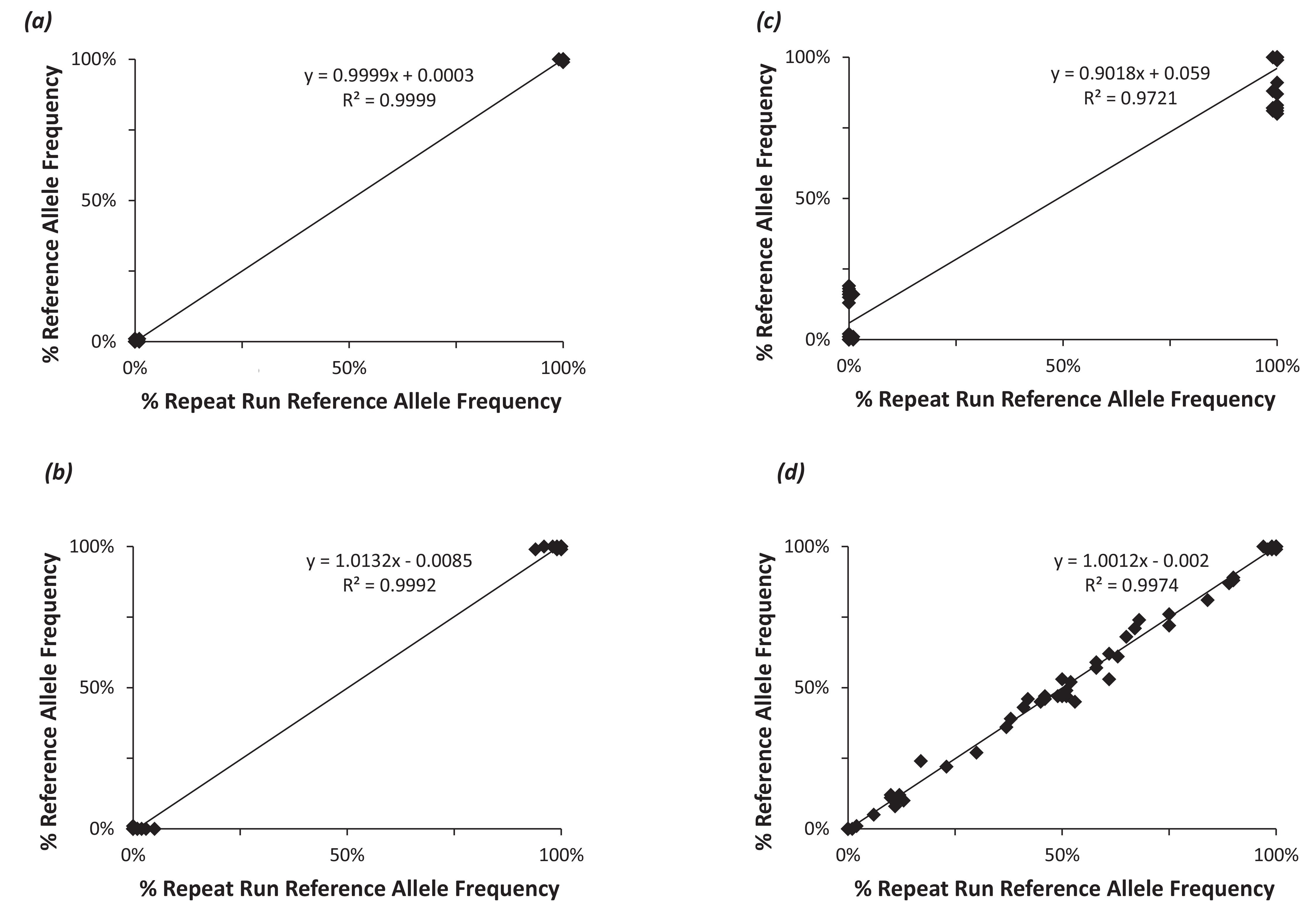

Supplement: S2 Fig — Each plot corresponds to a different infection, with monoclonal samples being shown in (a) and (b), and polyclonal in (c) and (d). Each diamond represents one SNP and is displayed according to its allele frequency in the duplicated samples. (TIF) [file pntd.0004526.s004.tif]

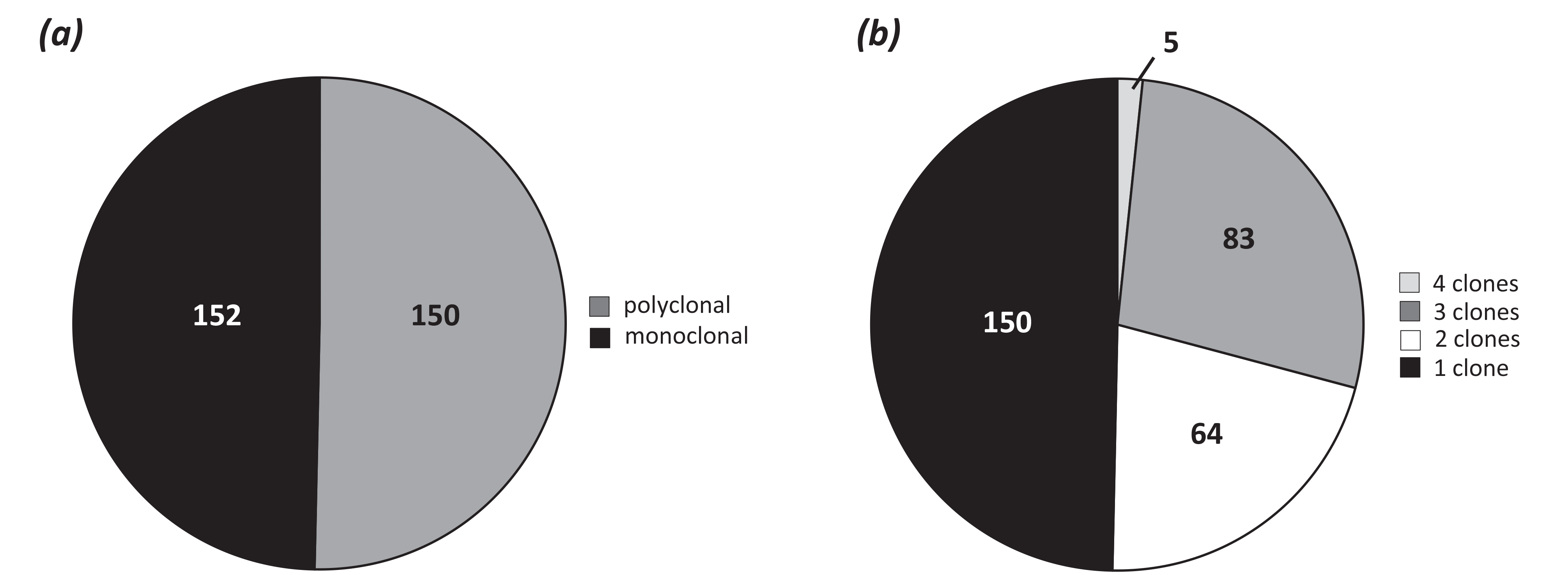

Supplement: S3 Fig — The infections are separated in monoclonal and polyclonal infections (a) and according to the most likely number of clones (b). Numbers indicate sampling size for each category. (TIF) [file pntd.0004526.s005.tif]

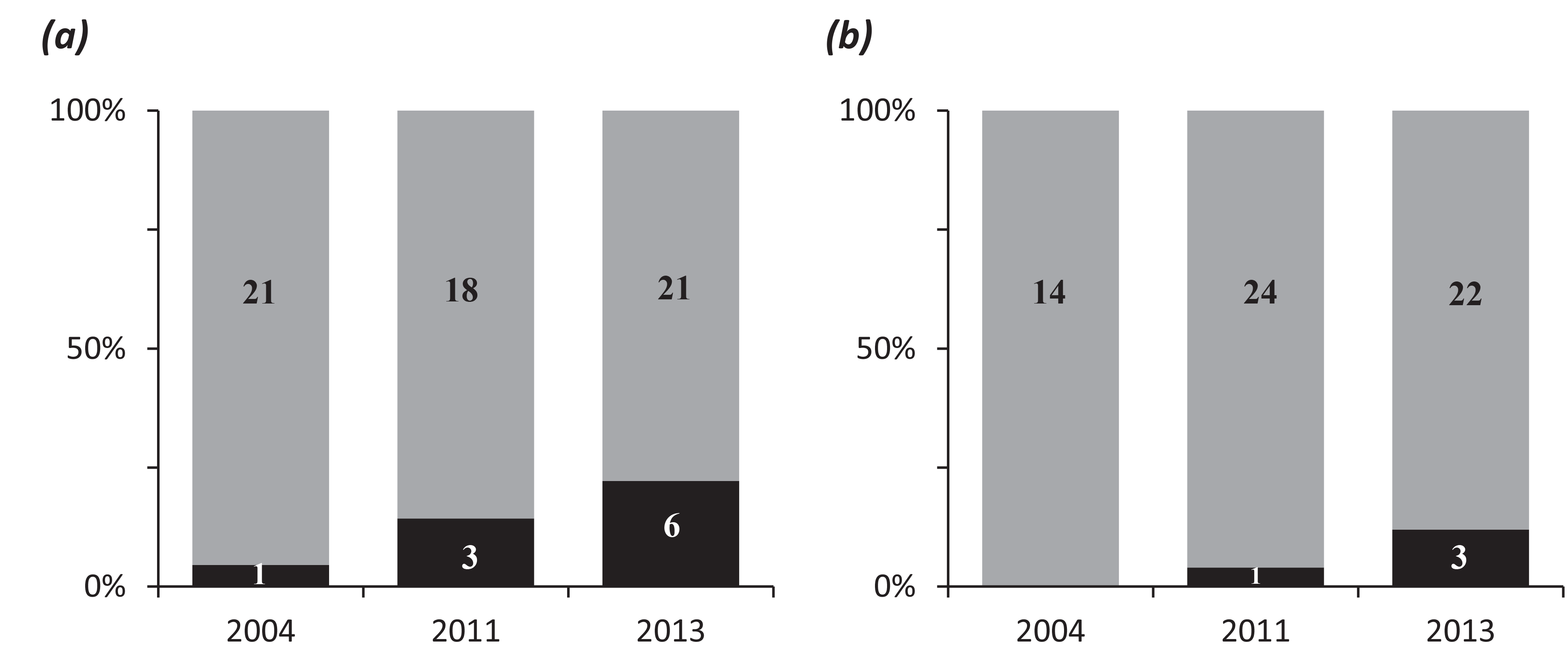

Supplement: S4 Fig — The proportion of monoclonal infections is shown in black and the polyclonal infections in grey. The figures indicate the actual number of samples in each category. (TIF) [file pntd.0004526.s006.tif]
